# Supplementary material for: Global hypo-methylation in a proportion of glioblastoma enriched for an astrocytic signature is associated with increased invasion and altered immune landscape
Source: eLife. 2022 Nov 22;11:e77335. doi: 10.7554/eLife.77335 (PMC9681209; doi:10.7554/eLife.77335)
Supplement: Figure 2—source data 1. [file elife-77335-fig2-data1.zip › Figure_2_source_data_1/Figure_2I_J/homerResults/motif56.similar.html]

motif56

## Information for motif56

T
A
G
C
A
G
C
T
A
G
T
C
G
A
T
C
A
T
G
C
A
G
T
C
A
G
T
C
G
T
C
A
A
G
T
C
A
G
T
C
A
G
T
C
G
A
T
C
G
A
T
C
G
A
T
C
T
A
G
C
A
G
T
C
A
G
T
C
G
A
T
C
T
G
A
C
G
A
C
T
A
T
G
C
G
T
C
A
A
T
G
C
T
A
G
C
G
T
A
C
  
Reverse Opposite:  

A
C
T
G
A
T
C
G
T
A
C
G
C
A
G
T
T
A
C
G
C
T
G
A
A
C
T
G
C
T
A
G
C
T
A
G
T
C
A
G
A
T
C
G
C
T
A
G
C
T
A
G
C
T
A
G
C
T
A
G
A
C
T
G
T
C
A
G
C
A
G
T
T
C
A
G
T
C
A
G
T
A
C
G
C
T
A
G
C
T
A
G
T
C
G
A
A
T
C
G
  

|  |  |
| --- | --- |
| p-value: | 1e-15 |
| log p-value: | -3.649e+01 |
| Information Content per bp: | 1.577 |
| Number of Target Sequences with motif | 33.0 |
| Percentage of Target Sequences with motif | 4.55% |
| Number of Background Sequences with motif | 2.5 |
| Percentage of Background Sequences with motif | 0.90% |
| Average Position of motif in Targets | 102.0 +/- 51.9bp |
| Average Position of motif in Background | 153.5 +/- 2.8bp |
| Strand Bias (log2 ratio + to - strand density) | 1.4 |
| Multiplicity (# of sites on avg that occur together) | 1.45 |
| Motif File: | file (matrix) reverse opposite |

### Similar de novo motifs found

|  |  |  |  |  |  |  |  |
| --- | --- | --- | --- | --- | --- | --- | --- |
| Rank | Match Score | Redundant Motif | P-value | log P-value | % of Targets | % of Background | Motif file |
| 1 | 0.649 | T C A G T C A G T C A G T C A G T C A G T C A G T C A G T C A G T C A G T C A G T C A G T C A G T C A G T C A G T C A G T C A G T C A G T C A G T C A G T C A G T C A G T C A G T C A G T C A G T C A G T C A G T C A G T C A G T C A G T C A G T C A G T C A G T C A G T C A G T C A G T A C G T C A G T C A G T C A G T C A G T A C G T A C G T C A G T A C G T A C G | 1e-9 | -21.960328 | 2.48% | 0.48% | motif file (matrix) |
| 2 | 0.648 | A T G C A G T C A G T C A T G C A G T C A G T C A G T C A G T C A G T C A G T C A G T C A G T C A G T C A G T C A G T C A G T C A G T C A G T C A G T C A G T C A G T C A G T C A G T C A G T C A G T C A G T C A G T C A G T C A G T C A G T C A G T C A G T C A G T C A G T C A G T C A G T C A G T C A G T C A T G C A G T C | 1e-8 | -19.986919 | 2.34% | 0.62% | motif file (matrix) |
| 3 | 0.696 | C T A G G C A T A G T C G A T C C T G A A G C T A T G C C G T A A G C T G A T C G A T C A G T C G T A C A G T C T C G A A G C T A G T C G A T C T G C A G A C T | 1e-7 | -18.071016 | 2.20% | 0.34% | motif file (matrix) |
